# Focus characterization at an X-ray free-electron laser by coherent scattering and speckle analysis

**DOI:** 10.1107/S1600577515004361

**Published:** 2015-04-14

**Authors:** Marcin Sikorski, Sanghoon Song, Andreas Schropp, Frank Seiboth, Yiping Feng, Roberto Alonso-Mori, Matthieu Chollet, Henrik T. Lemke, Dimosthenis Sokaras, Tsu-Chien Weng, Wenkai Zhang, Aymeric Robert, Diling Zhu

**Affiliations:** a Linac Coherent Light Source, SLAC National Accelerator Laboratory, 2575 Sand Hill Road, Menlo Park, CA 94025, USA; b Deutsches Elektronen-Synchrotron, Notkestrasse 85, D-22607 Hamburg, Germany; c Institute of Structural Physics, Technische Universität Dresden, D-01062 Dresden, Germany; d Stanford Synchrotron Radiation Lightsource, SLAC National Accelerator Laboratory, 2575 Sand Hill Road, Menlo Park, CA 94025, USA; e PULSE Institute, SLAC National Accelerator Laboratory, 2575 Sand Hill Road, Menlo Park, CA 94025, USA

**Keywords:** X-ray FEL, speckle, focusing

## Abstract

X-ray focus optimization and characterization based on coherent scattering and quantitative speckle size measurements was demonstrated at the Linac Coherent Light Source. Its performance as a single-pulse free-electron laser beam diagnostic was tested for two typical focusing configurations. The results derived from the speckle size/shape analysis show the effectiveness of this technique in finding the focus’ location, size and shape. In addition, its single-pulse compatibility enables users to capture pulse-to-pulse fluctuations in focus properties compared with other techniques that require scanning and averaging.

## Abstract 2

An application of speckle size analysis for characterizing the focus at a free-electron laser source is presented. The performance of the speckle-based beam focus diagnostic is also compared with other common focus characterization techniques.

## 1. Introduction   

The recent advent of X-ray free-electron laser (FEL) sources has and continues to create enormous opportunities for modern scientific research (Emma *et al.*, 2010 ▶; Ishikawa *et al.*, 2012 ▶). In many ways the experiments using X-ray FELs are capitalizing on the decades of experience gained from those conducted at synchrotron sources around the world. However, the extreme peak brightness of an FEL combined with its unique timing structure and the stochastic nature of the self-amplified spontaneous emission (SASE) lasing process introduces many new experimental challenges. One of the areas that require special attention is beam diagnostics. Unlike X-ray beams generated by synchrotron sources which are extremely stable over the course of a typical experiment, all parameters of the X-ray FEL beam fluctuate, some rather significantly, from one pulse to another. Therefore, pulse-to-pulse monitoring is crucial for understanding the properties of the FEL processes as well as enabling data filtering and sorting, which is a critical requirement for refined data analysis.

In comparison with intensity, position, timing and spectral diagnostics developed over the past few years at the two current operating X-ray FELs (Feng *et al.*, 2011 ▶; Tono *et al.*, 2011 ▶; Harmand *et al.*, 2013 ▶; Lemke *et al.*, 2013*a* ▶; Bionta *et al.*, 2014 ▶; Zhu *et al.*, 2012 ▶; Inubushi *et al.*, 2012 ▶), beam focusing diagnostics have made relatively limited progress (Moeller *et al.*, 2011 ▶). However, a major fraction of experiments performed at FELs require some degree of X-ray beam focusing, using either Kirkpatrick–Baez (KB) mirrors, compound refractive lenses (CRLs), or zone plate diffractive optics. The reasons and subsequent requirements for focusing the X-ray beam vary from experiment to experiment. High-field physics experiments very often require producing the smallest possible focus to achieve the highest photon fluence (Young *et al.*, 2010 ▶). Pump–probe experiments typically prefer matching the optical pump and X-ray probe volumes, which very often are limited by the available pump pulse energy and therefore a tightly focused pump beam (Chollet *et al.*, 2015 ▶) is used. X-ray photon correlation spectroscopy (XPCS) experiments require a small scattering volume so that the resulting details of the coherent scattering pattern (*i.e.* speckle pattern) can be well resolved by X-ray detectors with relatively large pixel sizes (Grübel *et al.*, 2008 ▶; Shpyrko, 2014 ▶). Serial femtosecond crystallography seeks optimal matching between the sizes of the beam and the crystals being injected (Boutet *et al.*, 2012 ▶).

There are several well established focusing characterization techniques currently used at X-ray FELs. Among them the most commonly implemented at the Linac Coherent Light Source (LCLS) are imaging using scintillator screens, knife-edge scans, imprints and the recently developed ptychography method, as briefly described below.

*Imaging*. A high-resolution microscope to optically image the X-ray induced fluorescence on a thin (<100 µm) scintillator screen is widely used at the LCLS for beam profile diagnostics. It produces direct images of the beam profile for individual pulses, allowing the measurement of pulse-to-pulse fluctuations of the beam position and shape. This method routinely achieves a 3–5 µm spatial resolution. It is a convenient way to locate the beam, but lacks the resolution to reveal the actual size and shape of a tight focus.

*Knife-edge scans*. A knife-edge scan (Bilger & Habib, 1985 ▶) or wire scan (Fulton *et al.*, 1989 ▶) is the most frequently used method because of the simplicity of its setup. During a knife-edge scan a sharp edge is translated precisely through the beam while the total transmitted intensity is recorded using a single-element detector downstream (Cannon *et al.*, 1986 ▶). With the setup stability well taken care of, resolution well below 1 µm has been achieved for both synchrotron source and the X-ray FELs (Mimura *et al.*, 2010 ▶, 2014 ▶; Yumoto *et al.*, 2013 ▶). One disadvantage, however, is that this method is by nature a scanning technique and one-dimensional. It measures only the projection of the average beam profile along the direction perpendicular to the scanning direction. Its validity relies on the assumptions that the beam profile has a simple shape and that the stability between the beam and the scanning setup is much better than the size of the beam. Focus position and shape fluctuations tend to result in an overestimation of the beam size. In addition, locating the focus requires multiple scans at different locations and in two orthogonal directions, making the technique relatively time-consuming.

*Imprints.* The imprint technique provides excellent spatial resolution as shown in recent works by David *et al.* (2011) ▶ and Yumoto *et al.* (2013) ▶. However, a quantitative understanding requires electron microscopy of a large number of craters as well as accurate measurements of the corresponding pulse intensities (Liu, 1982 ▶). It is also time-consuming and is not well suited as a real-time feedback for focus optimization. Moreover, although each imprint can be created by a single shot, the interpretation of the results is statistical in nature and thus requires a large number of measurements.

*Ptychographic reconstruction.* A recent focus characterization work using ptychography achieved few-nanometer resolution and produced a full three-dimensional view of how the X-ray wavefront propagates through the focus for both the amplitude and the phase (Schropp *et al.*, 2013*a* ▶). This method is based on scanning coherent diffraction microscopy, and conveniently takes advantage of the full transverse coherence of the FEL beam. The setup and algorithm are both being developed towards a real-time focusing diagnostics (Schropp *et al.*, 2013*b* ▶). Nevertheless, it shares the disadvantage with the imprint and the knife-edge techniques that being a scanning technique it requires a large number of measurements. As such, shot-to-shot variations in the focus properties will not be captured. The reconstruction, albeit showing superb spatial resolution, reflects the properties of a statistical average.

The discussion above indicates that, for an X-ray FEL, a focus diagnostic which can provide pulse-to-pulse details beyond one dimension is highly desired. In this article we demonstrate that by performing coherent scattering measurements using well understood samples, speckle size/shape analysis can be used as a viable alternative to provide a single-pulse diagnostics for rapidly locating the focus, obtaining a first estimate of the focal spot size, and capturing potential focus position, size and shape fluctuations.

## 2. Coherent diffraction and speckle size   

Speckles, generally referring to the granularity of intensity observed when optical coherent light reflects off a rough surface (Goodman, 2007 ▶), have found numerous applications in the X-ray regime as the coherent properties of X-ray sources kept improving. The most prominent examples are X-ray coherent diffractive imaging (Chapman & Nugent, 2010 ▶) and XPCS (Grübel *et al.*, 2008 ▶; Shpyrko, 2014 ▶). In the forward-scattering geometry, the far-field intensity spatial distribution of the coherent scattered X-rays (*i.e.* the speckle) in the detector plane is related to the Fourier transform of the X-ray wave in the sample plane following the Fraunhofer diffraction formalism.

Given a sample with granular phase contrast, little absorption, and sufficient spatial resolution from the detector, a first observation is that the far-field speckle size is inversely proportional to the illumination volume, *i.e.* the beam size. The larger the speckle, the smaller the beam size is at the sample, and thus the closer the sample is to the focus. The relation between the speckle ‘area’ and the scattering spot size was discussed and derived in detail by Goodman (2007 ▶). To first order, the size of the speckles 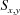 is related to the beam size on the sample plane 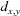 by 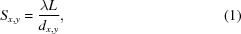
where *L* is the distance between the sample and the detector, and λ is the wavelength. In practice, a correction factor is needed to reach a more accurate estimate for the beam size, which depends on the properties of the scatterers as well as the exact shape of the beam. This is because the size interpretation essentially relies on deducing the width of a function from its autocorrelation. For our case we introduced an adjustment factor α for the beam size expression: 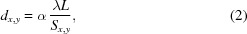
and calibrated α through numerical simulations. Based on sample dimensions, well known optical constants and the assumption of a Gaussian beam profile, we arrived at 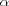 = 0.85 ± 0.05.

Moreover, as the scattering and measurement geometry is fundamentally two-dimensional, the reduction to a one-dimensional ‘size’ can be arbitrary. While in this paper we limit our discussion mainly to the horizontal and vertical sizes, the two-dimensional intensity autocorrelation of the speckles can contain a lot more information, *e.g.* when it has a tilted elliptical shape, or has multiple side lobes.

## 3. Focus characterization by speckle analysis   

**Figure 1:**
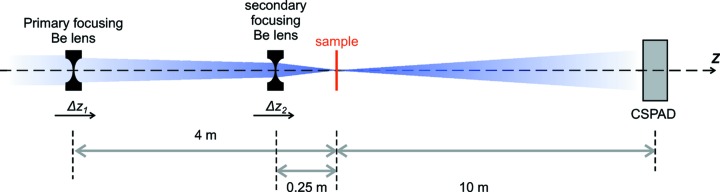
Schematic of the focusing setup used at the XPP instrument. The X-ray beam is focused by either a single set of beryllium compact refractive lenses nominally located 4 m upstream of the sample (labeled as ‘primary focusing’) or by two sets of lenses installed 4 m and 0.25 m upstream of the sample (‘primary’ and ‘secondary’ focusing). Both lenses are installed on motorized stages to allow translation along the beam propagation direction by 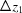 and 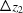, respectively.

In this section we present speckle analysis obtained at the X-ray Pump Probe (XPP) instrument (Chollet *et al.*, 2015 ▶). The optical layout for the measurement is shown in Fig. 1 ▶. The incident X-ray energy was 8.2 keV. A Si(111) monochromator was used to define the bandwidth and minimize chromatic aberrations. Beryllium CRLs were installed at two locations: 4 m and 0.25 m upstream of the sample location. At the 4 m location, two CRL lens sets were available: one with a focal length of 4 m, and another with a focal length of 8 m. The 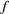 = 4 m set had an effective radius of 40 µm and a diffraction-limited focal spot size of ∼1 µm. The 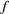 = 8 m set could be used to pre-focus the unfocused X-ray beam, which was 400 µm in size, in order to match the entrance aperture of the 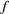 = 0.25 m CRL set. The 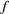 = 0.25 m set has an effective radius of curvature of 2.5 µm and has a larger numerical aperture, thus a tighter theoretical focus size of approximately 120 nm full width at half-maximum (FWHM). It consists of 20 beryllium CRLs with 50 µm radius of curvature having an effective aperture of 250 µm and a numerical aperture of 0.5 mrad. This 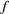 = 0.25 m set could be translated out of the beam when the 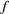 = 4 m set was used. Both lens sets can be translated along the beam direction *z* to allow focus location adjustment and optimization. A CSPAD-140k detector (Blaj *et al.*, 2015 ▶), positioned 10 m downstream of the sample location, was used to record small-angle coherent scattering patterns. A beam stop was positioned in front of the detector to block the direct beam.

A dried powder of 150 nm silica spheres in a 0.5 mm-diameter glass capillary was used as the scattering object. It was chosen because of the relatively strong scattering signal in the *Q* range that fits the size of the detector at a given distance, as well as its relatively high damage threshold. During the speckle measurements, the sample location was fixed and the lens set distances were varied to adjust the beam sizes at the sample location. For the single CRL set configuration we translated 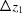 as indicated in Fig. 1 ▶. For the double CRL set configuration we keep 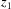 fixed at 4 m and translated 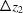.

**Figure 2:**
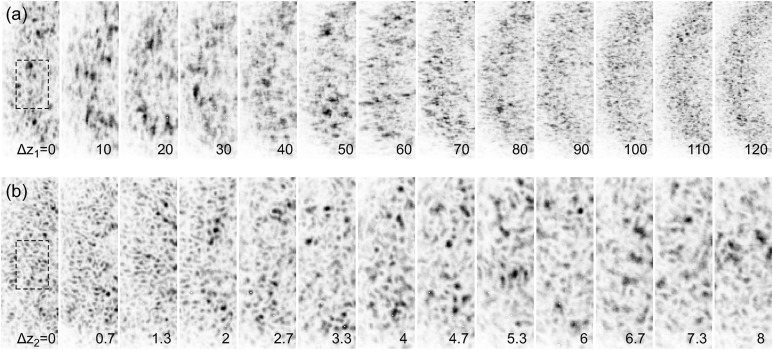
(*a*) Speckle patterns measured at different lens positions 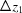 for the single CRL set configuration. (*b*) Speckle patterns measured at different lens positions 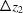 for the double CRL set configuration, with the position of the primary lens remained fixed at 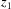 = 4 m. The shown portions of the scattering patterns are centered around 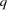 = 0.05 nm^−1^. All patterns are 120 shot (1 s) averages. Lens positions are indicated at the lower right-hand corners of each pattern in millimeters. The dashed rectangle in both 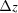 = 0 images indicates the region of interest used for subsequent speckle size analysis.

The superb transverse coherence of the beam led to the observation of very high contrast speckle patterns (Gutt *et al.*, 2012 ▶). The measured average speckle patterns as a function of 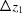 and 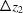 are shown in Fig. 2 ▶ for the two configurations. The observed speckle sizes and appearances varied significantly as *z* changed. As the speckle size becomes larger, it indicates that the focus is getting closer to the sample location. The visual appearance of the speckles can thus provide an immediate feedback for quickly locating the focal plane. We also observed distinct shapes of speckles from the two configurations. In the case of the single CRL set configuration, strong anisotropies of the speckles at 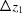 = 20 mm and 60 mm was a clear indication of astigmatism; *i.e.* the vertical focus is downstream of the horizontal focus. In the case of the double CRL set configuration, the appearance of the speckles is rather isotropic, indicating a less elliptical shape at the focus.

**Figure 3:**
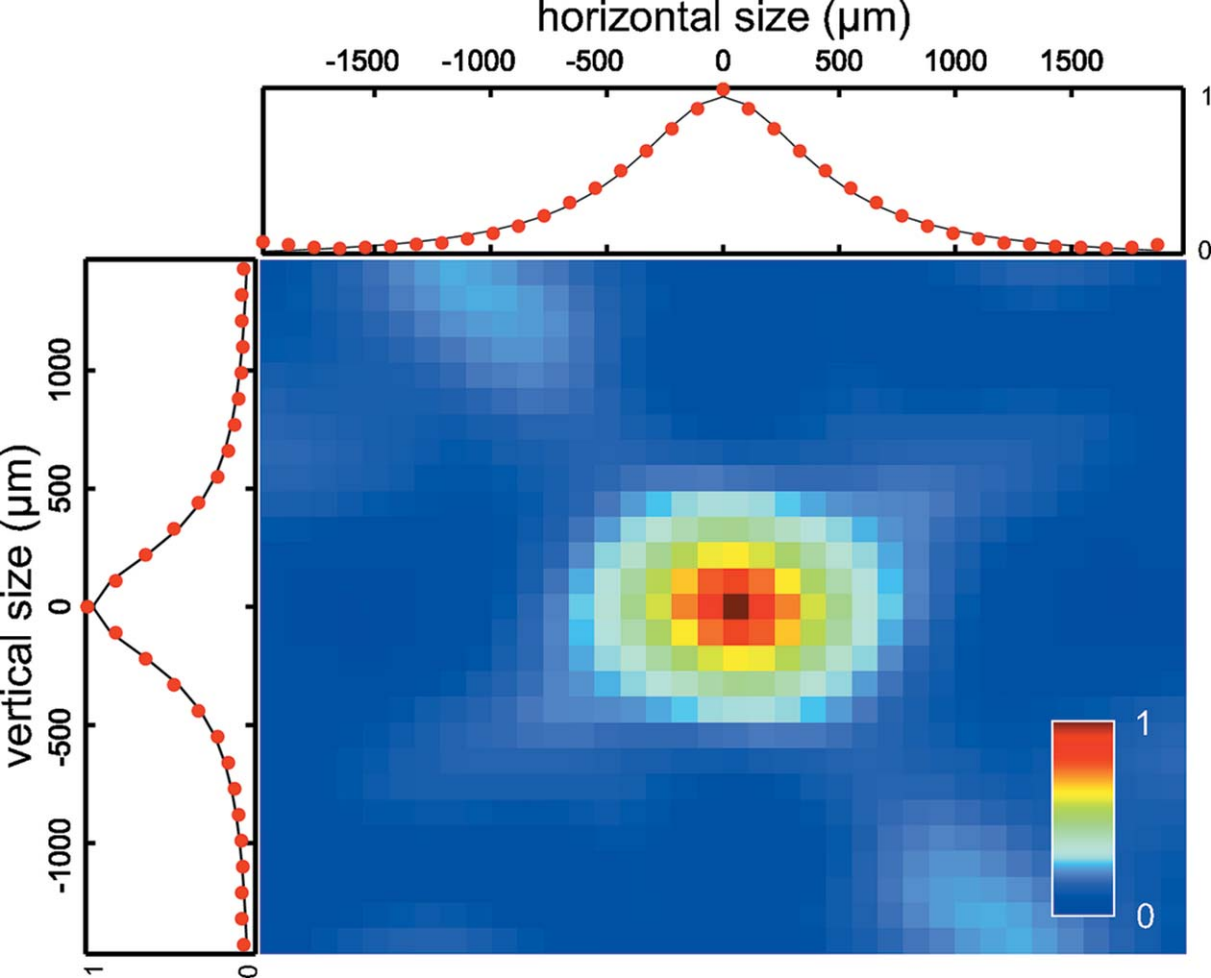
Calculated spatial autocorrelation function from a single-shot speckle pattern for the double CRL set focusing case at 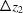 = 8 mm. Horizontal and vertical lineouts through the center of the autocorrelation and their Lorentzian fits are plotted around the image.

A quantitative analysis of the collected speckle patterns was then performed according to procedures commonly used for visible lasers as well as X-rays (Piederrière *et al.*, 2004 ▶). The spatial intensity autocorrelation function of each speckle pattern was calculated over a 30 × 50 pixel wide region of interest centered at wavevector transfer 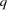 = 0.05 nm^−1^ matching the main structure factor peak of the silica powder, as indicated by the dashed rectangle box in Fig. 2 ▶. We applied Lorentzian fits to the vertical and horizontal lineouts of the two-dimensional autocorrelations. To obtain the final estimates for the speckle sizes 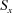 and 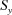, the FWHM of the fitted Lorentzian functions were used. An example of a two-dimensional spatial intensity autocorrelation and the central lineouts in both the horizontal and vertical directions are shown in Fig. 3 ▶. A small difference between 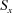 and 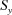 was observed.

**Figure 4:**
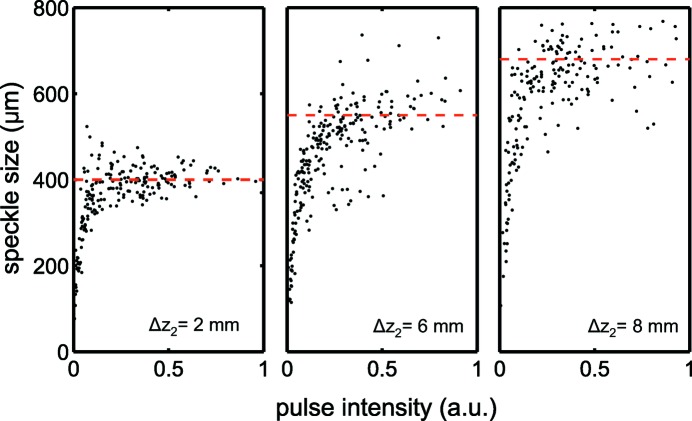
Calculated speckle size in the vertical direction *versus* pulse intensity for the double CRL focusing case at three different secondary lens positions 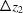. From left to right: 2 mm, 6 mm and 8 mm.

When evaluating speckle sizes from consecutive pulses, a large pulse-to-pulse variation was observed. Fig. 4 ▶ shows the calculated speckle sizes in the vertical direction *versus* the incoming pulse intensity for three different 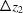 positions for the double CRL set configuration. The spread can be attributed to the pulse-to-pulse fluctuation in X-ray beam properties such as intensity and beam position, as well as profile. Another observation was that for low-intensity pulses the speckle size was systematically underestimated as a result of photon-counting noise. However, the flattening trend seen in all three plots indicates that the measurement converged to a good estimate with increasing pulse intensities. The remaining fluctuation in the calculated beam sizes can partially be attributed to real beam properties.

**Figure 5:**
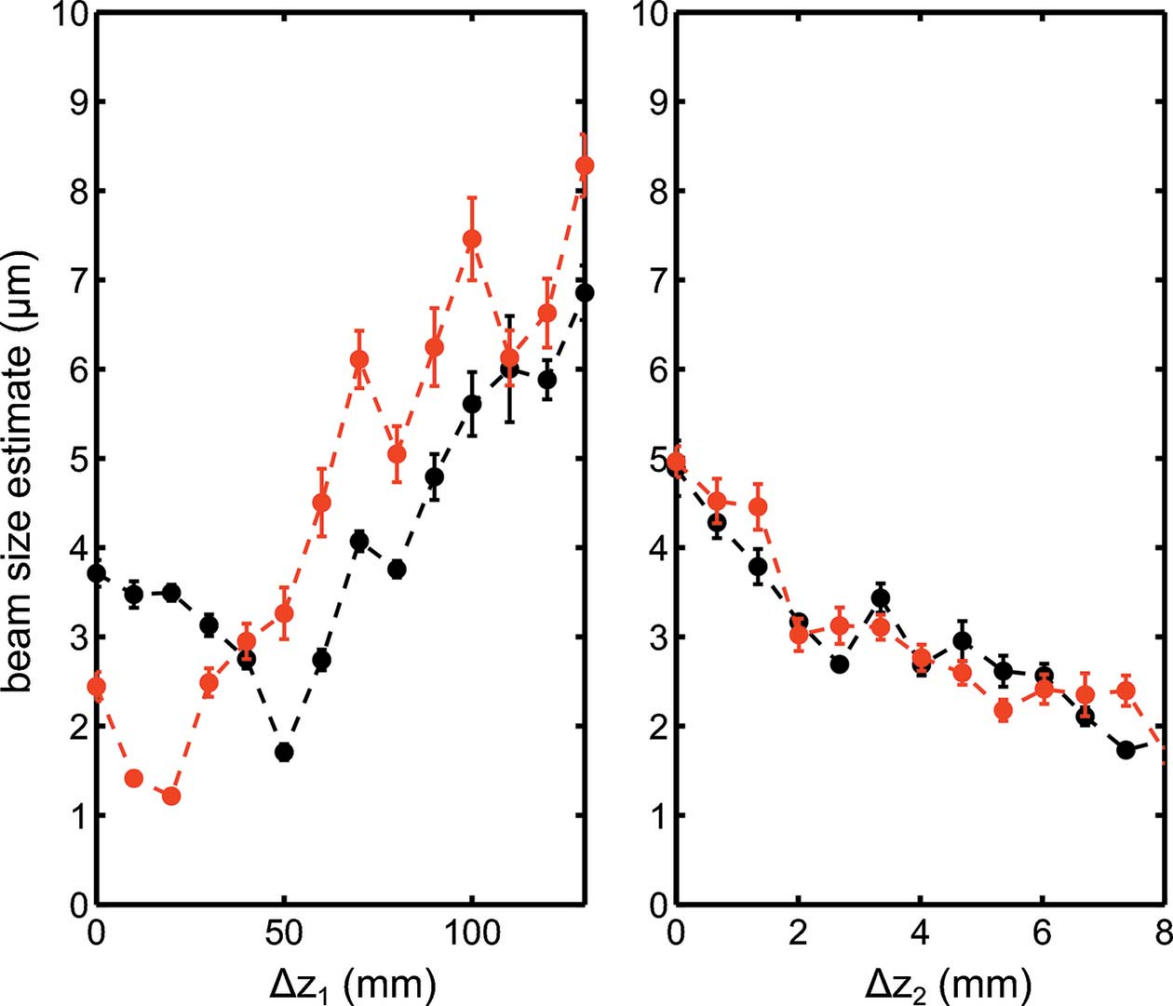
Derived beam sizes at sample location from speckle sizes, horizontal 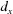 (black circles) and vertical 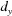 (red circles). Left: beam sizes *versus* 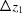 for the single CRL set focusing. Right: beam sizes *versus* 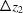 for the double CRL set focusing.

Finally, speckle sizes for the most intense pulses were averaged and converted into beam sizes 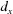 and 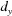 based on equation (2). As shown by the dashed red lines in Fig. 4 ▶, an average value of the pulses with intensities above 0.4 was used. Approximately 400 speckle patterns for the single CRL case and 150 speckle patterns for the double CRL case were used to generate the average size for each point. The results as a function of lens positions for both focusing configurations are displayed in Fig. 5 ▶. For the single CRL set configuration we observed that the vertical focus is about 30 mm downstream from the horizontal focus. In both directions the deduced beam sizes are slightly below 2 µm. For the double CRL set focusing case the focal spot size shows a monotonous decrease as 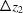 increases but never reaches below 1 µm. This is contrary to the focus location determined by both the Ronchi test (Nilsson *et al.*, 2012 ▶) and ptychography at approximately 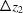 = 6.5 mm. We will discuss the discrepancy and the reason for the overestimation in the next section.

## 4. Comparison and discussion   

**Figure 6:**
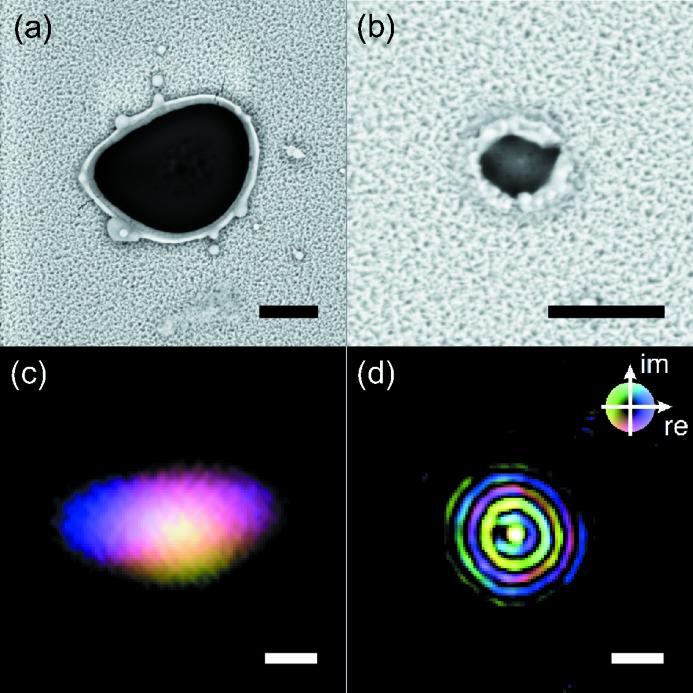
Beam focus measurement examples from imprints and ptychographic reconstructions. All scale bars are 1 µm in size. (*a*) Single CRL set focusing imprint. (*b*) Double CRL set focusing imprint. (*c*) Ptychographic reconstruction for single CRL set focusing. (*d*) Ptychographic reconstruction for double CRL set focusing. The color scale for both ptychographical reconstructions (*c*) and (*d*) are shown in the upper right-hand corner of (*d*), with the brightness and the color representing the amplitude and the phase, respectively.

The same experimental setup is readily compatible with imprint and ptychography techniques. We performed ptychography scans at 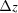 positions such that the target was slightly out of focus, based on the locations suggested by the speckle method. The imprints were then performed at the focus locations derived from ptychographic reconstructions. In the case of the single CRL set configuration, imprints were taken with 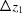 = 20 mm. In the case of the double CRL set configuration, 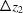 = 6.5 mm was used. Typical beam imprints and the final ptychographical reconstructions at those positions for both focusing configurations are shown in Fig. 6 ▶.

The measurements used the monochromatic beam. Pulse-to-pulse intensity variations led to large imprint size variations (Lemke *et al.*, 2013*b* ▶; Zhu *et al.*, 2014 ▶). The two single-shot imprints shown were among the smallest obtained on the target. We observed varying asymmetric shapes of the imprints, one of them shown in Fig. 6(*a*) ▶. This is a clear indication of the pulse-to-pulse focus shape fluctuation. The imprint in Fig. 6(*b*) ▶ provides direct evidence of a sub-micrometer central spot from the double CRL set configuration.

The ptychographical reconstructions shown in Figs. 6(*c*) and 6(*d*) ▶ are slices for 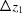 = 20 mm and 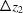 = 6.5 mm, respectively. The data sets were both obtained with 20 × 20 grid scans with 120 images (1 second) at each location. The convergence was rather robust against pulse-to-pulse intensity fluctuations after averaging. For the single CRL set focusing case, a focus size of 1.1 µm (V) × 1.8 µm (H) was given by the reconstruction at the corresponding 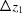. For the double CRL set configuration, the reconstruction reveals a central lobe 150 nm in size, surrounded by higher-order ring-shaped diffraction fringes. The rings were a result of diffraction from a 300 µm-diameter circular entrance aperture in front of the secondary CRL set. Spherical aberrations also contributed to this observation.

For the single CRL set configuration, the astigmatism was consistently observed by both imprint and ptychography at the correct 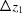 locations. The focal spot size estimation obtained from the different methods were also largely consistent.

For the double CRL set configuration, however, the estimation from the speckle analysis seems to reflect the size of the halos surrounding the central peak rather than the nanofocus central lobe. This is an indication that a significant portion of the photons reside in the first- and second-order rings, in partial agreement with ptychographical reconstruction which suggests approximately 80% of all the photons reside in the central 2 µm circular area of the beam profile.

On the other hand, the speckle results in this case completely missed the central lobe which contained approximately 15% of the photons. A main reason was the choice of the 150 nm nanoparticles, the size of which was too close to that of the central lobe size. It led to intensity modulations in *q* space similar to that of the resulting speckles. As a result, it becomes difficult to disentangle the contributions, thus preventing accurate quantification of the speckle size. To be sensitive to the smaller beam sizes and profile features, nanoparticles with sizes much smaller than the length scale of interest need to be used. In addition, the 0.5 mm-thick sample volume introduced smearing as well, because the speckle analysis provides information on the projected beam profile over that thickness, which is larger than the focal depth of a 150 nm focus. The use of a thinner sample would address this problem.

What makes speckle analysis based focus characterization an attractive alternative at X-ray FELs is its relatively simple setup and its ability to provide instant feedback. In all four LCLS hard X-ray instruments, infrastructures exist for small-angle coherent scattering measurements. At 10 m distance with the CS140 detector, the field of view of the diffraction geometry can resolve speckles from beam sizes up to 10 µm. Current detector development at LCLS will make available 50 µm pixel size X-ray detectors in the near future (Blaj *et al.*, 2015 ▶). This would effectively reduce the required detector distance by half. The scattering target can also be easily introduced to various sample environments. In cases where the sample for the experiment by itself produces high-contrast speckle patterns, no additional target is needed. In those cases, the beam size may even be monitored parasitically during the data acquisition. The calculation of 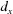 and 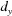 involves minimum computing resources and can be implemented to run during data acquisition.

## 5. Conclusion   

We demonstrated the application of coherent scattering as an effective focus optimization and characterization technique. Its compatibility with pulse-to-pulse operation gives it the unique advantage of capturing not only the average beam size and profile information but also pulse-to-pulse variations in the focus properties at X-ray FEL sources. The ability to provide instant feedback to the experiment makes it far more attractive than performing imprint measurements or knife-edge scans for locating the focus.

We envision more comprehensive analysis of speckle properties, especially in the form of temporal correlation analysis of consecutive speckle patterns, to reveal more details about the focus shape and positional stability, as well as their correlation with other machine parameters. With sufficient knowledge of the scatterer, *e.g.* by using a tailor-designed target, one can envision pulse-to-pulse phase retrieval that reconstructs the wavefront properties at the focus. For the relatively new field of X-ray FEL sciences, the ability to quickly diagnose and control the beam properties with increasing precision will finally enable more sophisticated experiments in the future.
